# High energy density solid state symmetric supercapacitors using ionic liquid dispersed Li^+^ ion-perovskites†

**DOI:** 10.1039/d4ra07417c

**Published:** 2025-01-27

**Authors:** Bhargab Sharma, Shrishti Sharma, Gurpreet Kaur, Hardeep, Anshuman Dalvi

**Affiliations:** a Department of Physics, BITS Pilani-Pilani Campus RJ-333031 India adalvi@pilani.bits-pilani.ac.in; b Department of Chemistry, BITS Pilani-Pilani Campus RJ-333031 India

## Abstract

The study reports solid-state ceramic supercapacitors (SSCs) assembled using a novel composite electrolyte based on Li^+^ ion conducting perovskite-type LLTO (Li_0.34_La_0.51_TiO_3_) and an ionic liquid (EMIM BF_4_). Small amounts of various ionic liquids (ILs) were added to LLTO to enhance the ionic conductivity and improve electrode compatibility. The optimal composition with approximately ∼6 wt% EMIM BF_4_ in LLTO exhibited a high ionic conductivity of around ∼10^−3^ Ω^−1^ cm^−1^ at room temperature, nearly three orders of magnitude higher than that of the pristine LLTO. Optimized electrolyte composition was therefore used for fabrication by compressing between high surface area activated carbon-coated copper electrodes and assembled in an affordable lamination cell geometry. The SSCs demonstrated stable cycling performance for at least 10 000 cycles at 2 V operating voltage and 1.13 A g^−1^ (2 mA) discharge current, with a remarkably high coulombic efficiency of ∼99%. A typical laminated cell at 35 °C exhibited a specific capacitance of around 510 F g^−1^ at 0.57 A g^−1^ (1 mA), and 2 V. Supercapacitors operating below 2 V showed a pure electric double-layer type nature. A stack of 4 cells in series can power two white LEDs (6 V) for ∼40 minutes.

## Abstract 2

The study reports solid-state ceramic supercapacitors (SSCs) assembled using a novel composite electrolyte based on Li^+^ ion conducting perovskite-type LLTO (Li_0.34_La_0.51_TiO_3_) and an ionic liquid (EMIM BF_4_).

## Graphical abstract

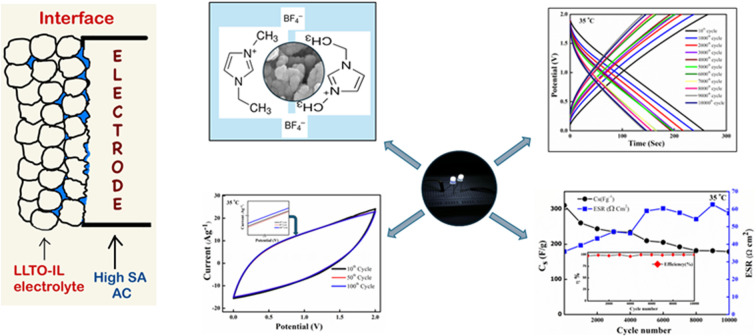

## 1 Introduction

Solid-state electrolytes have emerged as a crucial component in the development of next-generation energy storage devices, particularly in all-solid-state batteries (ASSBs).^1^ Unlike ASSBs, relatively less emphasis has been given so far to liquid-free solid-state supercapacitors (ASSCs). Even though the interface optimization for fast ionic movement is challenging, the solid electrolytes offer significant advantages, including improved safety,^2^ higher energy densities,^3^ and enhanced thermal stability.^4^ These characteristics make them particularly appealing for use in advanced Li^+^ ion and lithium-metal batteries, central to applications ranging from consumer electronics to electric vehicles.^5^ Along with Li^+^ ion solid-state batteries, the demand for solid-state supercapacitors has increased in recent years due to their extensive and low-dimensional and high-temperature applications.^6^ Significant attention has been given to fast ionic ceramics, *viz.* Li^+^/Na^+^ NASICONs, garnets, and perovskite structured systems. Despite the high in-grain conductivity of these ceramics, practical application is tricky due to high grain boundary impedance (GBI) and the poor interface with electrodes.^7–9^ Tailoring the grain boundary interface by adding some fillers (ionic liquids) can dramatically improve the overall conductivity of the system.^10^

ASSBs use solid ionic conductors, also known as solid-state electrolytes (SSEs). These include composite solid polymers,^11^ inorganic (*e.g.*, ceramic-based oxide electrolytes), and hybrids of inorganic/organic systems.^12^ However, poor solid–solid interfacial contacts limit the practical applications of solid ionic devices. Various strategies have been suggested to overcome the electrode–solid electrolyte contact issues.^13^ Gel polymer electrolytes (GPE), have also drawn attention due to their high ionic conductivity (≥10^−3^ Ω^−1^ cm^−1^), relatively better interfacial contact and lower risk of flammability than the liquids, substantially higher electrochemical voltage window and increased flexibility.^14–18^ Applications are, however, limited as they have poor mechanical strength and a narrow operating temperature range.^19^ It is now realised that if the impedance at grain boundaries, and electrode–electrolyte interface is improvised the fast ionic ceramics could be the best choice for electrolytic applications.

One method for tailoring the interface involves adding a small amount of ionic liquid to the ceramics. Hayashi *et al.* reported CSP 75Li_2_S·25P_2_S_5_ glass with the addition of ionic liquid 10 mole% of 1-ethyl-3-methyl imidazolium bis(flourosulfonyl)amide with lithium bis(trifluromethanesulfonyl)amide showed a high conductivity of 10^−3^ Ω^−1^ cm^−1^ at room temperature.^20^ Further, Rathore *et al.* conducted a study on the effect of adding the ionic liquid (BMIM BF_4_) to Li^+^ ion oxide glass and their glass ceramics and revealed that adding a small amount of ionic liquid (0.5–5 wt%) significantly increased the ionic conductivity. For a typical glass composition of 60Li_2_SO_4_-40(0.5Li_2_O-0.5P_2_O_5_), dispersing about 5 wt% of ionic liquid resulted in a conductivity increase of around 2–4 orders of magnitude.^21^ In an exciting work, Nowiński *et al.* reported a LiTi_2_(PO_4_)_3_ and Li_1.3_Al_0.3_Ti_1.7_(PO_4_)_3_ based composites containing 2–10 wt% of 1-butyl-3-methylimidazolium tetrafluoroborate (BMIM BF_4_) ionic liquid with a high total ionic conductivity at room temperature.^22^ Kaur *et al.* have recently reported the synthesis of LiTi_2_(PO_4_)_3_ composites dispersed with EMIM BF_4_ IL through the sol–gel method, resulting in conductivity ranging from 10^−3^ to 10^−4^ Ω^−1^ cm^−1^.^23^ In a subsequent study, the same type of NASICON was doped with Al^3+^ to enhance conductivity to ∼10^−3^ Ω^−1^ cm^−1^. Supercapacitors were fabricated using these materials, and approximately 10 000 cycles of charge and discharge were performed with almost 100% efficiency.^24^ Kaur *et al.* recently prepared garnet composites with ionic liquid Li_6.75_Al_0.25_La_3_Zr_2_O_12_ (LALZO) to improve its ionic conductivity. The optimal composition of ∼3–6 wt% ionic liquid in LALZO showed a high ionic conductivity of 6 × 10^−4^ Ω^−1^ cm^−1^ at room temperature.^25^ These were applied as electrolytes in 2032-type Li/LiCoO_2_ button-type cells and were stable under battery conditions.

Perovskite (ABO_3_) lithium lanthanum titanate and its derivatives have also been demonstrated to be promising Li^+^ ion conducting electrolytes. The structure of this material contains a significant number of ‘A’ site vacancies created by the disordered arrangement of the Li^+^ and La^2+^ ions, which facilitates lithium-ion transport. To migrate from one A site to the next, available Li^+^ ions must pass through a bottleneck of four surrounding oxygen atoms.^26^ In 2002, Nalini *et al.* Reported that the conductivity of LLTO perovskite improved with the Eu^+^ substitution on the ‘B’ site. Since then, several efforts have been made to improve the total ionic conductivity of the LLTO by doping with materials such as LiF, LLZO, Sr, Ba, Ca, Ta, ZrO_2_, LiO_2_–SiO_2_–B_2_O_3_, Ag, and Ge. The enhancement of total conductivity through doping can be attributed to the improved bulk conductivity, grain boundary conductivity, or both. However, the improvement of total conductivity has not yet met the expectations for commercial lithium-ion electrolytes.^27^ It has been found that amorphous LLTO-like compounds could exhibit high Li^+^ conductivity. These materials are typically prepared as films using the sol–gel method or PVD. For instance, the conductivity of amorphous film is 9.56 × 10^−6^ Ω^−1^ cm^−1^ compared to 0.64 × 10^−6^ Ω^−1^ cm^−1^ for fully crystallized film.^28^ Despite exhibiting high ionic conductivity and stability in a wide temperature range, their potential has not been explored so far in supercapacitors. For the recent few years, we have attempted to apply these fast ionic ceramics as electrolytes with their tailored interface using ILs. Using such IL-added ceramics leads to the development of thermally stable and mechanically more robust supercapacitors.^29–31^

Due to various promising properties related to electrolytic applications it was felt important to explore Li^+^ ion perovskites for applications to supercapacitors. Herein, we have developed a hybrid material by combining LLTO with an ionic liquid in a small amount (1–8 wt%). The role of IL is kept to a minimum when examining the LLTO potential in supercapacitors. This hybrid material exhibits a room temperature conductivity of 10^−3^–10^−4^ Ω^−1^ cm^−1^. Various structural and thermal characterization techniques have been performed to test the performance of this mixed material as an electrolyte in solid-state supercapacitors (SSCs) and found it to be fast ionic. We used two different types of ILs, namely EMIM BF_4_ and EMIM CF_3_SO_3_, to synthesize the corresponding composites with LLTO. Our work addresses the critical issues related to the fabrication and characterization of IL-LLTO-based SSCs. We evaluated the role of IL and Li^+^ ions from the LLTO framework in the device performance. Significantly, these hybrid composites differ from ionogels,^32^ as the amount of IL added is small (∼6 wt%).

Our work attempts to identify the factors that affect the performance of these ceramic supercapacitors and rectify them to get performance parameters comparable to those of liquid/gel-based supercapacitors.

## 2 Experimental

The perovskite-type Li_0.34_La_0.51_TiO_3_ (LLTO) has been synthesized by using the sol–gel route.^33^ A stoichiometric amount of LiNO_3_ and La(NO_3_)_3_·6H_2_O is added to ethylene glycol monomethyl ether for complete dissolution stirring at ∼35–40 °C for 1 h. To the above solution, titanium(iv) butoxide and acetylacetone are added in a 1 : 1 ratio, and the final solution is stirred at 60 °C for ∼6 h to obtain a brownish gel-like mixture, which was heated at 150 °C for around 2 h. The black-white compound obtained was crushed and further calcined at 900 °C for 6 h. The resulting white LLTO powder was obtained afterward. The LLTO–IL composites were prepared by mixing the LLTO powder uniformly with IL in a planetary ball mill (Fritsch-P6) for 1 h in an agate pot with a sample mass ratio of 5 : 1. Two different ionic liquids were used for making composites *viz.* EMIM BF_4_ and EMIM CF_3_SO_3_ in (1–8 wt%) ratio is used for composite preparation. The samples were labelled as LLTO–*x*IL, where *x* is the weight percentage of IL varies from 1 to 8%. One could add up to 8% of the IL while keeping the composite homogeneous. Beyond this intake, the IL separates out as excess liquid. Most measurements were carried out for composite containing an optimum ∼6 wt% IL in the LLTO matrix.

X-ray diffraction measurements (XRD) were carried out for these LLTO–IL-based composites using a Rigaku Miniflex II X-ray diffractometer with CuKα radiation (*λ* = 1.54 Å). Field emission scanning electron microscopy (FESEM) with the FEI-Apreo-S instrument was used to investigate the surface morphology of the composites. A transmission electron microscope (TEM) with JEOL JEM-F200 was used to observe the lattice fringes and the polycrystalline nature of LLTO. The Thermogravimetric Analysis (TGA) measurements were performed using the DTG-60 series (SHIMADZU) at 10 °C min^−1^, in the 30–500 °C temperature range under a nitrogen atmosphere. X-ray photoelectron spectroscopy (XPS) (Thermoscientific K-α) was also used to comprehend charge states, oxidation states, and the elemental composition of the LLTO and LLTO–IL composites prepared. The surface areas of the LLTO, LLTO–6IL, and LLTO–10IL were measured using a BET surface area analyzer, BELSORP-MINI X. The electrical conductivity under steady-state conditions was determined using the HIOKI IM3570 impedance analyzer over a wide frequency (4 Hz–5 MHz) and a temperature range (40–165 °C). The composites were pressed at ∼5 T to make 9 mm diameter pallets LLTO with *x* wt% of IL is abbreviated as LLTO–*x*IL. After applying conductive graphite paint to the surface, the pellets were kept at approximately 100 °C for 2 hours. The cells were then sandwiched between stainless steel electrodes for electrical transport measurements.

### 2.1 Supercapacitor fabrication

**Fig. 1:**
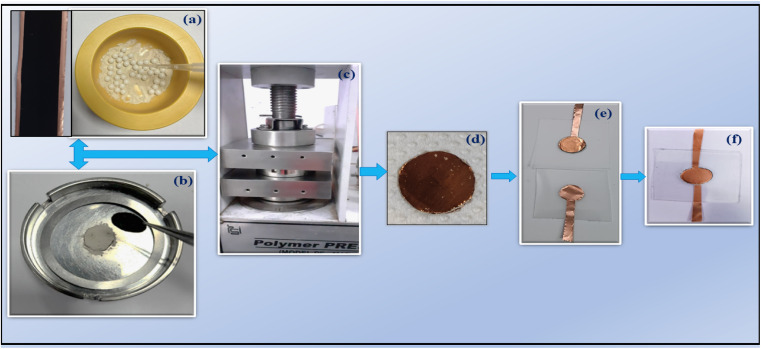
Fabrication process steps for SSC having activated carbon (AC) coated copper foil and IL dispersed LLTO composite. IL–LLTO composite layer of ∼0.5 mm thickness was used as an electrolyte. (a) Initial mixing of LLTO and IL in a planetary ball mill (b) placing the composite powder between AC-coated electrodes (c) pressing the cell with ∼3 tons per cm^2^ in a hydraulic press at room temperature for ∼10 minutes (d) the as fabricated supercapacitor (e) laminated cell components (f) final laminated supercapacitor for characterization.

The LLTO–*x*IL composites were further tested under supercapacitor conditions. A high surface area (∼2000 ± 100 m^2^ g^−1^) of activated carbon was used for the electrode preparation. The electrode slurry preparation, coating, and drying process to obtain the final electrodes have been followed as described earlier.^24^ The mass loading per electrode was ∼1.2 mg cm^−2^, and electrodes (diameter ∼14 mm) were cut into circular shapes for device fabrication. The LLTO–6% EMIM BF_4_ composite electrolyte was placed between electrodes and put under pressure of up to ∼4 tons per cm^2^ in a hydraulic press. Then, this sandwiched configuration consisting of electrolytes of various composites in between electrodes was transferred to a laminated supercapacitor geometry, as shown in Fig. 1. To characterize these SSCs, different techniques such as electrochemical impedance spectroscopy (EIS), cyclic voltammetry (CV), and galvanostatic charge–discharge (GCD) were carried out using electrochemical workstation Autolab 204. Fig. 1 shows the fabrication process of the supercapacitor in laminated cell geometry.

## 3 Results and discussion

### 3.1 Structural and thermal studies

#### 3.1.1 XRD analysis

**Fig. 2:**
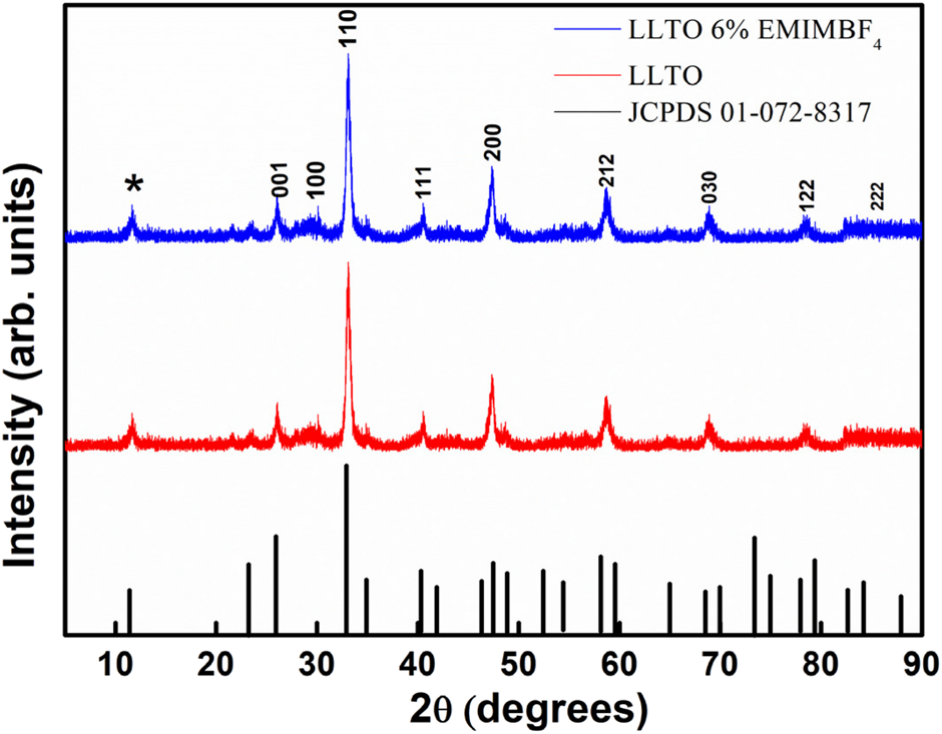
XRD pattern for pristine LLTO and LLTO–6IL (JCPDS card number 01-072-8317).

The XRD patterns for Pristine LLTO and LLTO + 6% EMIMBF_4_ (LLTO–6IL) pellet are shown in Fig. 2. The marked peaks corresponding to the JCPDF data in the inset matches well with the obtained peaks. The (Li_0.34_La_0.51_TiO_3_) exhibits a perovskite structure, and some of the peaks were found to have emerged due to the superlattice structure, as indicated by an asterisk (*).^33^ The crystallite size for LLTO is found to be ∼34 nm using Debye Scherrer relation. No new peaks or significant peak shifts were observed after adding the ionic liquid. This suggests that ionic liquid addition does not lead to any reaction leading to precipitation of crystalline compounds. Further, the ionic liquid does not enter into the lattice of the LLTO; it stays in the grain–grain interface. This readily suggests that the LLTO–IL composite is stable.

#### 3.1.2 TGA analysis

**Fig. 3:**
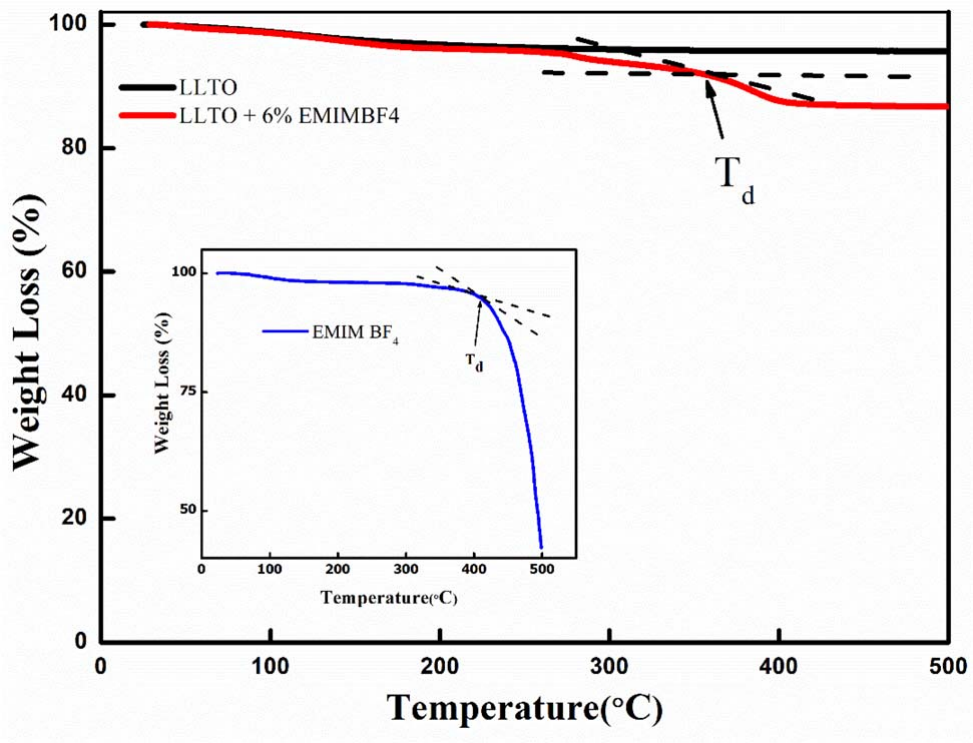
TGA plots of LLTO compared with LLTO–IL. (Inset) TGA plot for pristine EMIMBF_4_.

TGA plots of LLTO compared with LLTO–IL composite and EMIMBF_4_ (inset) are shown in Fig. 3. Pristine LLTO does not exhibit any abrupt weight loss when the temperature rises. Still, there is a gradual loss from 100% to 95% in a temperature rising from room temperature to 500 °C. The LLTO's water content could cause this slight weight reduction. Again, the presence of various organic residues left over after the LLTO sol–gel process may cause a prolonged and gradual weight reduction. It is readily seen that the decomposition temperature for the LLTO–IL composites is around 360 °C, which appears before that of the pristine IL, around 400 °C (inset Fig. 3). Since the IL along with the water partially adsorbs on the LLTO, this weight loss may be attributed to desorption process which is prolonged and gradual. This suggests in the composites, the IL is not bound by a unique, strong chemical bond but held up by the adsorption of different magnitudes.

#### 3.1.3 TEM and FESEM studies

**Fig. 4:**
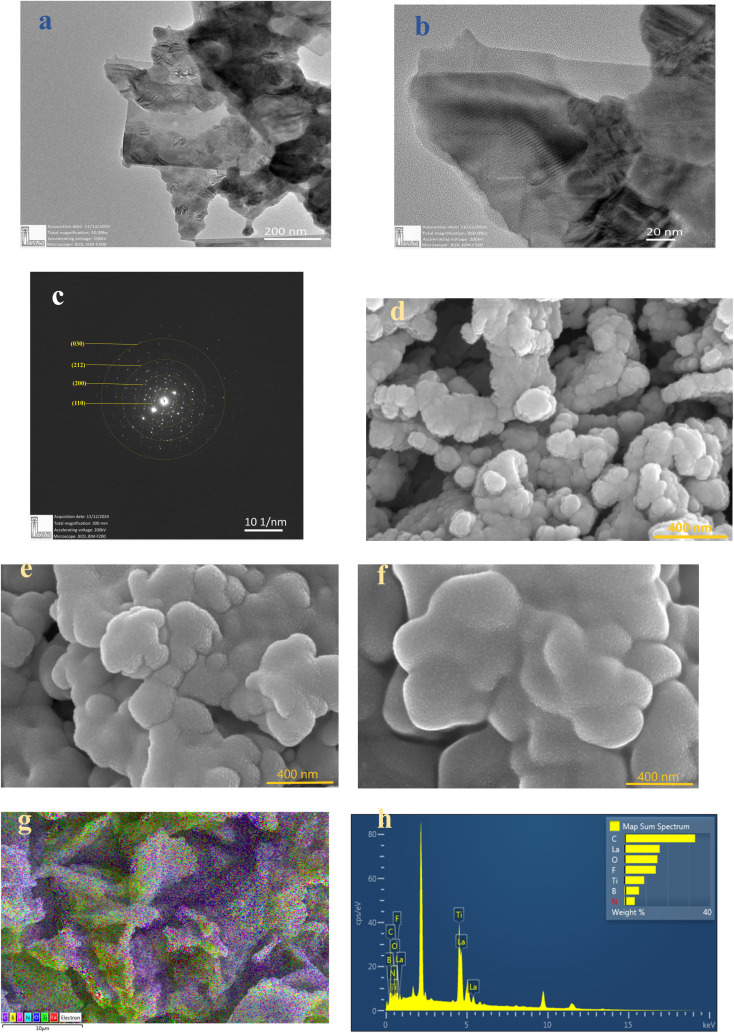
TEM images of (a) LLTO (b) lattice fringes of LLTO (c) SAED of LLTO. FESEM images of pristine (d) LLTO (e) LLTO–6%IL and (f) LLTO–10% IL (g) EDX with colour spectrum (h) EDS spectrum of LLTO–6IL electrolyte at 10 μm.

The microstructure of the pristine LLTO was studied using TEM analysis. Fig. 4(a) displays the TEM image of LLTO, highlighting the polycrystalline nature of the ceramic particles, where various grains are clearly visible. Lattice fringes can be apparently observed at a scale of 20 nm, Fig. 4(b). The polycrystalline structure of LLTO is further confirmed by the Selected Area Electron Diffraction (SAED) pattern in Fig. 4(c), which aligns with the planes obtained from the XRD patterns.

Fig. 4(d)–(f) present FESEM images of pristine LLTO, alongside comparisons with LLTO–6%IL and LLTO–10%IL. The grains are of uniform size with no signs of aggregation. There is no noticeable reduction in grain size after adding the ionic liquid, indicating that LLTO does not degrade at the interface with the ionic liquid. Fig. 4(g) shows the colour mapping of LLTO–6IL, revealing the presence of ionic liquid between LLTO grains. The IL does not segregate but shows an even distribution in the LLTO matrix. The Energy Dispersive Spectroscopy (EDS) spectrum shown in Fig. 4(h) also confirms that impurities are negligible. Moreover, the grains do not swell, indicating that the ionic liquid does not diffuse into the grains of LLTO but only remains at the grain boundaries or interparticle interface.

#### 3.1.4 XPS analysis

**Fig. 5:**
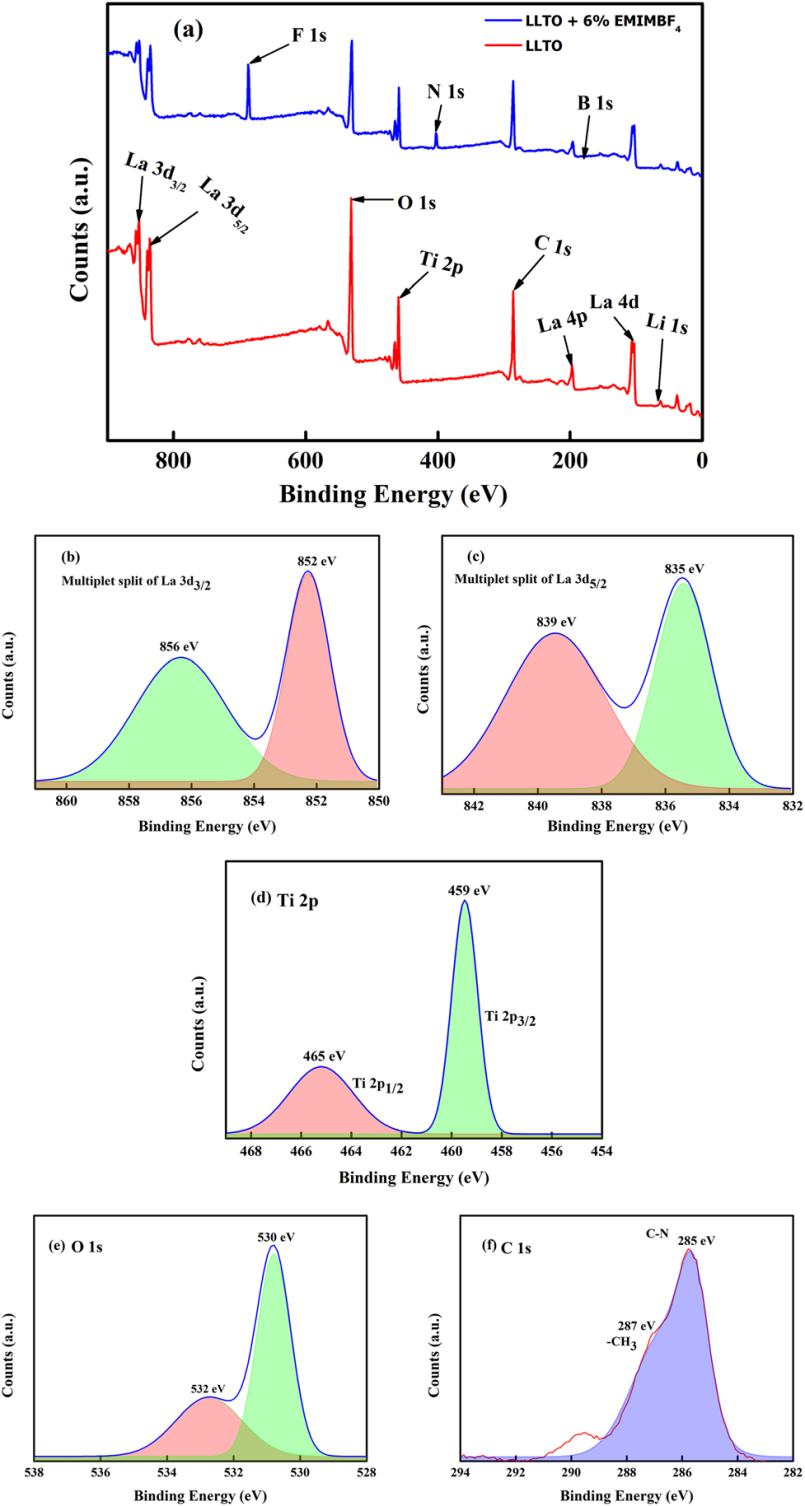
(a) Survey spectra of LLTO and LLTO IL deconvoluted peak of (b) La 3d_3/2_ (c) La 3d_5/2_. Deconvoluted peaks of (d) Ti 2p (e) O 1s (f) C 1s.

Fig. 5(a) and (b) shows the survey spectra of LLTO and LLTO + 6% EMIM BF_4_. All the elemental peaks are visible at their respective binding energies. A representative Ti 2p deconvoluted photo spectrum is depicted in Fig. 5. It closely matches the oxidation state of Ti^4+^ coordinated with six O_2_^−^ ions as seen in TiO_2_. The doublet is a result of spin–orbit coupling. The separation between the Ti 2P_1/2_ peaks is approximately 5.7 eV, and the area ratio is close to 2 : 1, consistent with findings from other studies.^34^ The full width at half maximum (FWHM) for each spin–orbit component is typically the same. However, for the Ti 2p, the Ti 2p_1/2_ component is much broader than the Ti 2p_3/2_ peak. As a result, the Ti 2p_1/2_ peak is much shorter than expected. On the other hand, most studies on titanate perovskites have pointed out that the Ti 2p_3/2_ peak exhibits a small shift towards lower binding energy (BE) compared to Ti^4+^ in TiO_2_. This shift is attributed to the presence of the cation, which influences the covalent character of the Ti–O bonding, resulting in an increase of the bond length.^35^ The spectrum of La 3d_5/2_, as shown in Fig. 5(b), can be fitted with two peaks separated by about ∼4 eV. There is a general agreement in the literature on X-ray photoelectron spectroscopy (XPS) that the double peak structure of each spin–orbital component of La 3d reflects states with the configuration 3d^9^4f^0^L and 3d^9^4f^1^L, where L denotes the oxygen ligand, and underscoring denotes a hole.^36^ The La 4p and La 4d regions are found at 196 eV and 103 eV, respectively. Since La is highly reactive, it is usually found in an oxidized state. The O 1s core level spectrum of the LLTO is depicted in Fig. 5(e), showing two peaks: one at approximately 530 eV, which is characteristic of titanate oxygen, and another at 532 eV, attributed to hydroxyl species. From the survey spectra of LLTO + 6% EMIM BF_4_, the deconvoluted C 1s photo spectrum reveals the presence of C–N species of the imidazolium ions at 285 eV, while CH_3_ groups of the IL and/or hydrocarbons originating from surface contamination are situated at 287 eV.^37^ All the other peaks B, F, Li, *etc.*, are observed at their respective binding energie. It is very astonishing to note that after addition of ionic liquid in LLTO there is no visible shifting of the BE peaks moreover the present peaks of the elements like Li, La, Ti, O *etc.* are not suppressed. This readily suggests that ionic liquid does not play any major surface alteration in the LLTO, and it just remain there as a mixture. Again, the Ti element exist in valence state of Ti^4+^.

#### 3.1.5 LLTO–IL BET surface area analysis

**Fig. 6:**
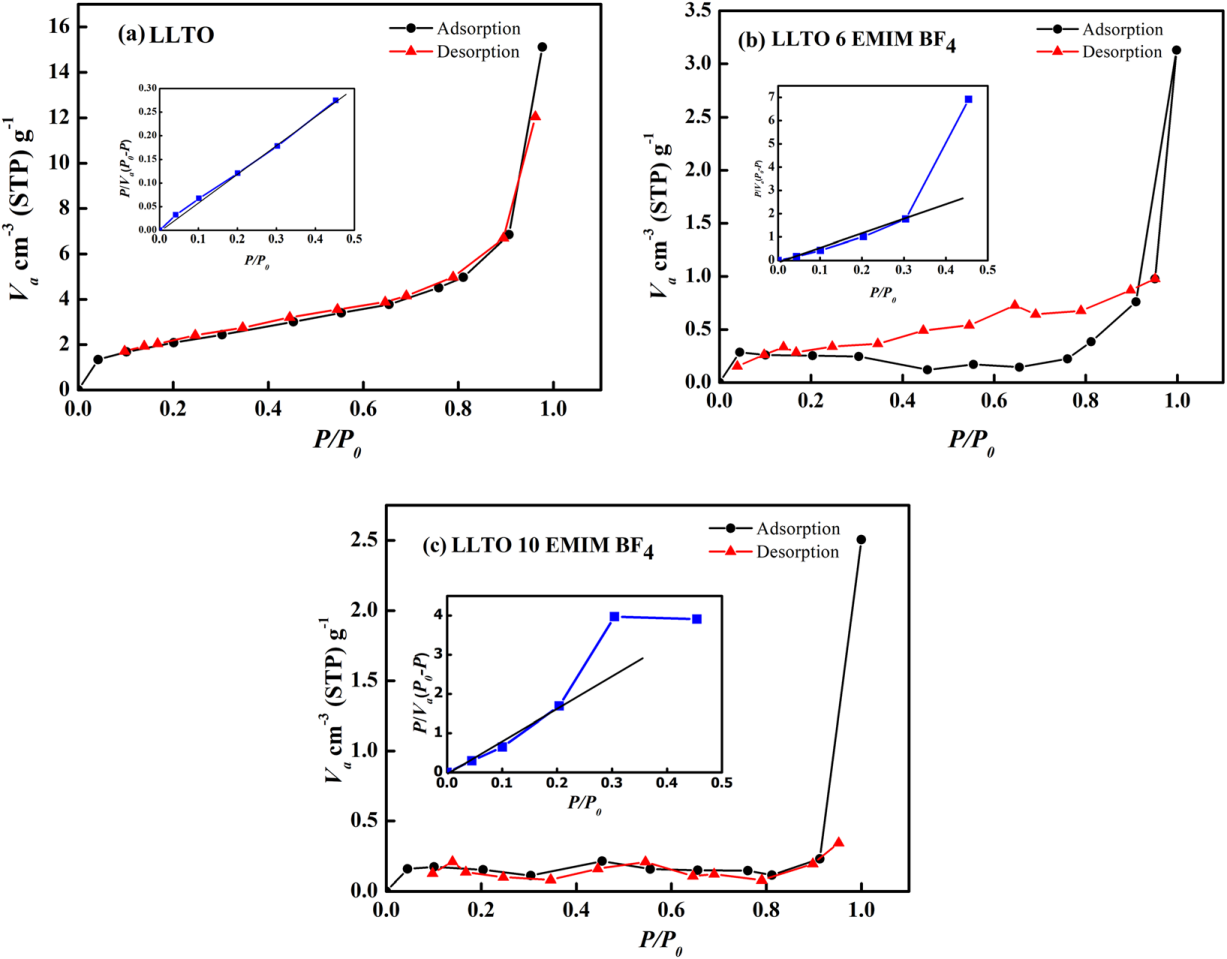
Nitrogen adsorption–desorption isotherm of (a) LLTO Inset shows linear fitting with BET equation. (b) LLTO 6 EMIM BF_4_. Inset shows linear fitting with BET equation. (c) LLTO 10 EMIM BF_4_. Inset shows linear fitting with BET equation.

The surface area (SA) of the LLTO electrolyte, before and after adding ionic liquid was analyzed using the Brunauer–Emmett–Teller (BET) technique.^38^ Thus, the SA of LLTO, LLTO–6IL and LLTO–10IL were obtained and compared. For this analysis, nitrogen adsorption isotherms were obtained at 77 K using a BET apparatus, as shown in the accompanying Fig. 6(a). The LLTO exhibited a low surface area, resulting in only a minimal increase in nitrogen uptake, with a very low volume of nitrogen adsorbed at relative pressures below 0.1. In contrast, at higher relative pressures (0.1 to 1), a noticeable plateau was observed along with a very small nitrogen uptake, which may be attributed to significant microporosity and narrow pore size distribution. From the inset of Fig. 6(a), the surface area of the LLTO was evaluated using the BET equation, yielding a value of approximately 9 m^2^ g^−1^. Fig. 6(b) and (c) show the adsorption isotherms for LLTO–6IL and LLTO–10IL, respectively. The surface areas for LLTO–6IL and LLTO–10IL, as derived from the insets of Fig. 6(b) and (c), were found to be ∼1 m^2^ g^−1^ and 0.5 m^2^ g^−1^, respectively. It is noteworthy that upon the addition of the ionic liquid, the nitrogen adsorption decreased, indicating a lower volume of nitrogen was adsorbed. Furthermore, the surface area of the LLTO decreased substantially by ∼89% and 96% after adding 6% and 10% IL, respectively in the LLTO matrix. This suggests that the ionic liquid occupied the pores and space between grains and expected to also improve electrode–electrolyte interfacial contacts, which was otherwise not possible due to ceramic roughness of the LLTO ceramic.

### 3.2 Electrical transport

**Fig. 7:**
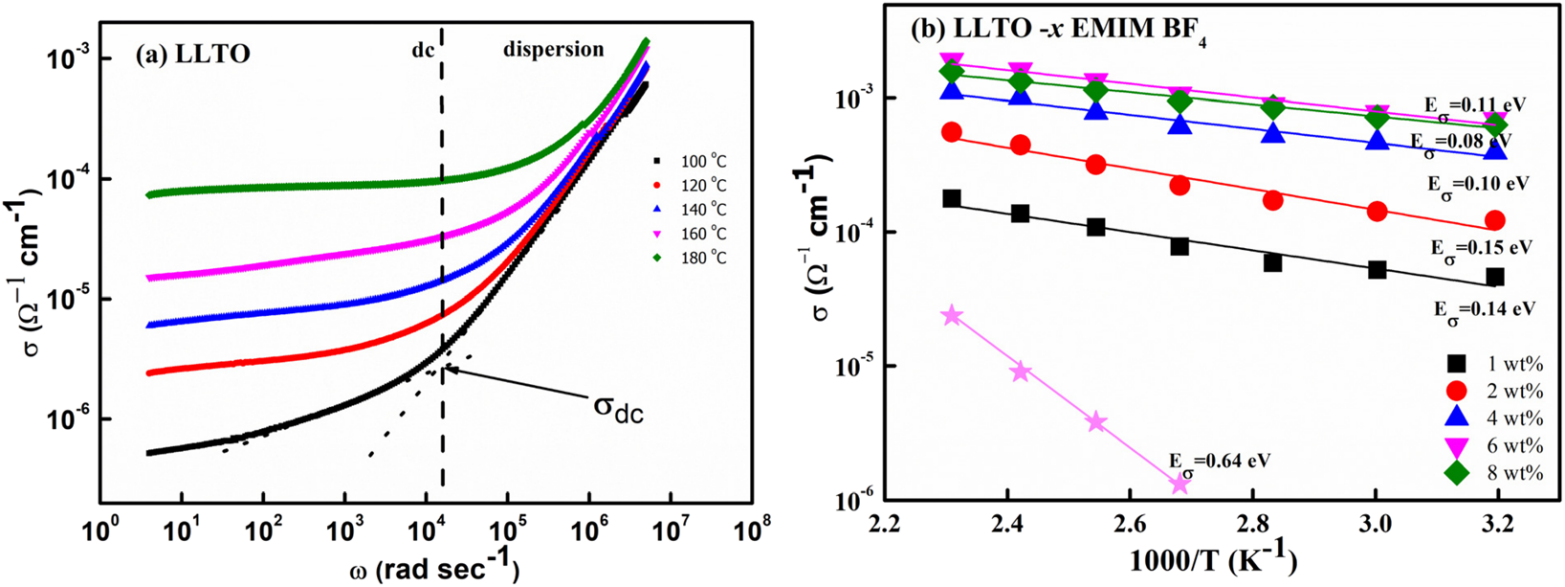
(a) Frequency dependence of electrical conductivity for pristine LLTO (b) temperature dependence of total conductivity for all the composites having LLTO and varied amount of EMIM BF_4_.

At the outset, the steady-state electrical conductivity was measured and plotted as a function of frequency. The DC conductivity was obtained from the plateau region *i.e.*, from the onset point of dc-dispersion as marked by the dotted lines as shown for the pristine LLTO (Fig. 7(a)). As apparent, pristine LLTO conductivity at ambient temperatures exhibits a low value, (∼10^−6^ Ω^−1^ cm^−1^ at 100 °C) inadequate for device applications. It may be due to the high grain boundary impedance (GBI). The mobile ion faces obstacles during long diffusive motion while passing from one grain to another. The bulk conductivity in such polycrystalline systems is generally high but due to large GBI its value is reduced by orders of magnitude,^39^ also reported for NASICONs^40^ and Garnets.^41^ However, after adding ∼1 wt% IL, the conductivity of the system increases by at least two orders of magnitude, as evident in Fig. 7(b). Further, the temperature dependence of the conductivity for IL–LLTO composites is also shown in Fig. 7(b) wherein the conductivity was obtained from the plateau of *σ*–*ω* plots (similar to Fig. 7(a)). Apparently, the conductivity exhibits linear (Arrhenius) behaviour with temperature. The cycles almost overlap for the compositions above ∼6% IL in LLTO; hence, the highest conductivity was witnessed for samples with 6 wt% IL. At room temperature, it surprisingly reaches to ∼10^−3^ Ω^−1^ cm^−1^ 40 °C.

**Fig. 8:**
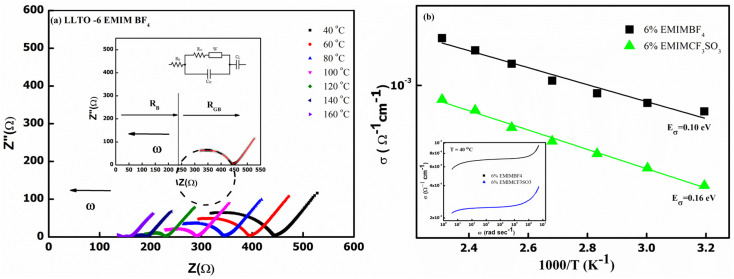
(a) Nyquist plot of the LLTO–6 EMIM BF_4_ with the equivalent circuit in the inset. (b) Total conductivity of LLTO with different IL. (Inset) Frequency dependence of the electrical conductivity of both LLTO–IL composite at 40 °C.

Fig. 7(b) also gives the activation energy (*E*_σ_) of the composite with varying (w/w)% of EMIM BF_4_. The *E*_σ_ values (0.11–0.14 eV) in comparison with that of pristine LLTO (∼0.64 eV) values are fairly low, suggesting a modest energy barrier for the ionic transport in IL presence. Interestingly, with the increase in the IL content, the activation energy monotonically decreases from ∼0.14 eV for LLTO–IL to ∼0.08 eV for LLTO–6IL, respectively. The LLTO–6IL gives the most optimised conductivity results. Fig. 8(a) shows the Nyquist plot with a corresponding equivalent circuit of the LLTO–6IL. It shows a depressed semicircle at a higher frequency followed by a tail at a lower frequency. For IL-added LLTO samples, the bulk, and the grain boundary contribution can be separated (Fig. 8(a)). The bulk and grain boundary resistance reduces with temperature. Further, the grain boundary impedance is manifested in the semicircle that is reduces notably due to IL presence. The interfacial polarisation is seen at a lower frequency is attributed to dominant ionic transport.

The type of ionic liquid was also found to be affecting the conductivity. Fig. 8(b) shows the conductivity trend of LLTO added with different types of IL. In both of these ILs, the cation size is kept the same. The size of the anion apparently plays a pivotal role in tailoring the conductivity of the LLTO. The inset of Fig. 8(b) shows the *σ*–*ω* plot of the above two composites, which again shows a higher conductivity for the case of LLTO–6 EMIM BF_4_. The composites with BF_4_^−^ ion, having a relatively smaller size of ∼0.205 nm than the CF_3_SO_3_^−^ ion of ∼0.203 nm, exhibit superior conductivity. The activation energy of the two systems compliments the above justification LLTO–6 EMIM BF_4_ composites have a relatively lower activation energy of ∼0.08 eV compared to the ∼0.16 eV of LLTO–6 EMIM CF_3_SO_3_.

The above results suggest that pristine LLTO has a higher grain boundary impedance, resulting in a large energy barrier for ionic transport IL addition substantially reduces these barriers. Additionally, EMIM BF_4_ is more suitable for preparing compositions used in device fabrication. The electrical transport is consistent with our earlier studies on IL–NASICON^24^ and IL–LALZO composites.^25^ However, in comparison to NASICON–IL composites, the required amount of IL for conductivity is relatively less.

The total conductivity may have the contribution of IL ions. Thus, the composites are suitable for supercapacitors where uni-ion motion is not required. The following section examines the LLTO–IL composites and their usage in supercapacitors. In the present system, the amount of ionic liquid is very low; thus, most mobile ions may be assumed from the LLTO matrix.

### 3.3 Electrochemical characterization

**Fig. 9:**
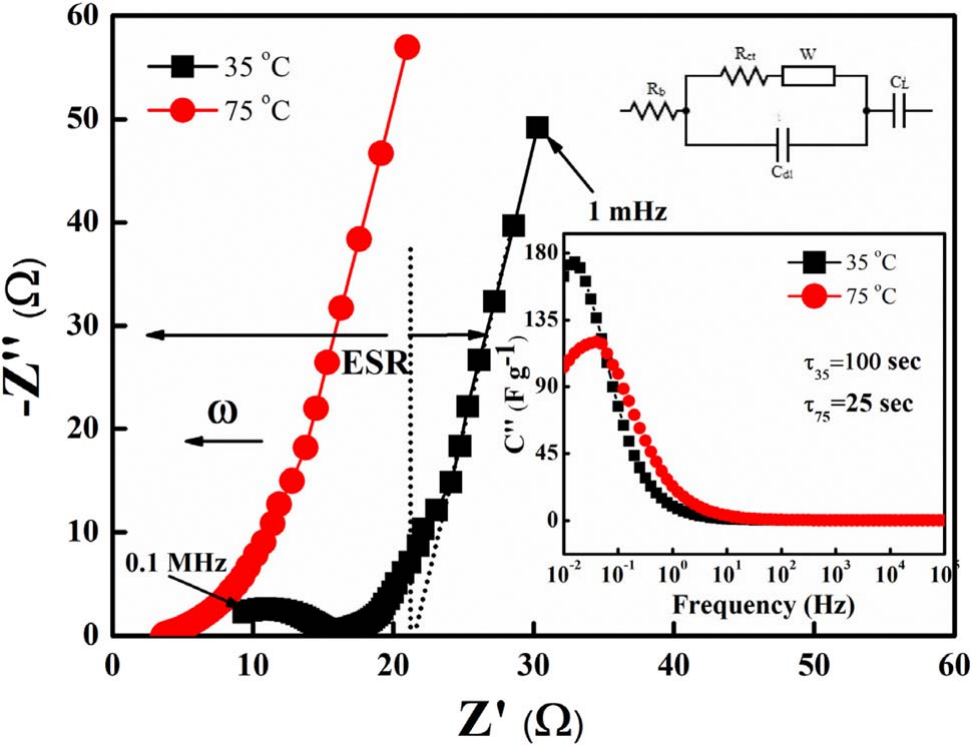
The Nyquist plot and corresponding equivalent circuit for the laminated geometry at two different temperatures having LLTO–6 EMIM BF_4_ composite as an electrolyte. (Inset) Imaginary capacitance (*C*′′) *vs.* frequency (Hz).

Firstly, the SSCs in laminated cell geometry were studied at 35 °C and 75 °C, respectively, to assess their wide temperature stability. At the outset, the Nyquist plots (1 mHz to 0.1 MHz) of the SSCs at two different temperatures are shown in Fig. 9. The behaviour provides apparent characteristics of a typical supercapacitor. The low-frequency inclined line with an almost vertical nature, the mid-frequency semicircular arc and the high-frequency termination on the real axis (*Z*′) confirm the formation of a supercapacitor. The mid-frequency semicircle of a small diameter corresponds to charge transfer at the interface, which suggests a possible faradaic process. However, the diameter of the semicircle is small; thus, the pseudo-process of the capacitance formation appears weak. The equivalent series resistance (ESR), the measure of the total internal resistance of the device, is obtained from the intersection of a vertical line with the *Z*′ axis, as shown by the dotted line.^42,43^ The ESR at 35 °C is found to be about ∼34 Ω cm^2^, which reduces to a value of ∼17 Ω cm^2^ at 75 °C. The impedance spectra apparently depict SSC behaviour. A model shown in Fig. 9 fits well with all these Nyquist plots. The inset of Fig. 9 shows the imaginary part of the capacitance (*C*′′) values 
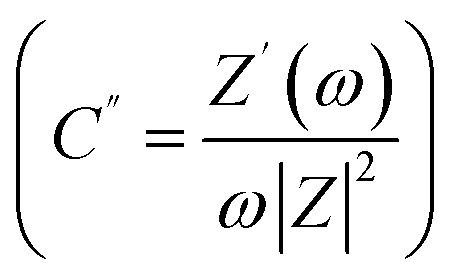
 which provides the dynamics of the charge storage process at the interface.^44^ As seen in the inset of Fig. 9, the *C*′′ *versus ω* apparently exhibits a relaxation that corresponds to the charge storage process in the electrode pores.^45^ The peak gradually shifts towards high frequency when the temperature increases which suggests that the mobile charges take a smaller time in reaching the pores for the capacitive action. Temperature rise increases the conductivity, thus activating the interfacial ion movement.

**Fig. 10:**
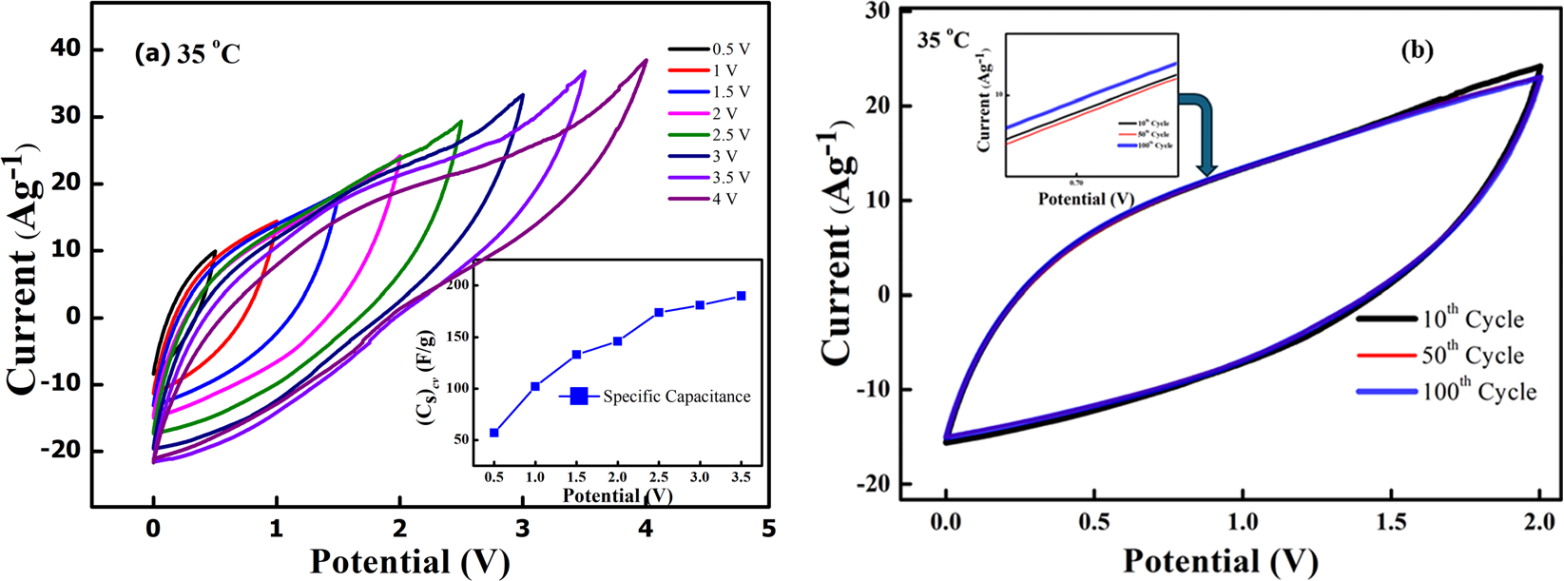
(a) CV scans (100 mV s^−1^) of SSC for different voltage range at 35 °C. (Inset) Variation of specific capacitance (*C*_s_)_cv_ with operating potential. (b) 100 cycles of CV scan (100 mV s^−1^) at 2 V and 35 °C.

In order to evaluate the working potential window of the electrolyte initially, the CV cycles and galvanostatic charge–discharge tests were carried out. Firstly, the CV patterns (100 mV s^−1^) at 35 °C were recorded for the device by gradually increasing the operating voltage range up to 4 V, as shown in Fig. 10(a). Apparently, the shape of the CV curve remains intact and almost featureless up to 3 V; above this a notable deviation is witnessed. For lower voltage, the curve is leaf-like. This may be due to low ionic mobility and movement.^46^ Charge storage ability at different voltages can again be depicted by observing the significant hysteresis in the CV. The Tafel slope analysis for Fig. 10(a) is presented in the ESI† that again complements dominant EDLC behaviour for operating voltage below 3 V. Fig. 10(b) shows the first 100 cycles (2 V, 100 mV s^−1^) at 35 °C. It is shown that up to 100 cycles, the device shows no degradation, and the electrochemical window of the device remains stable. The CV curves were also used to assess the specific capacitance per electrode using the formula 
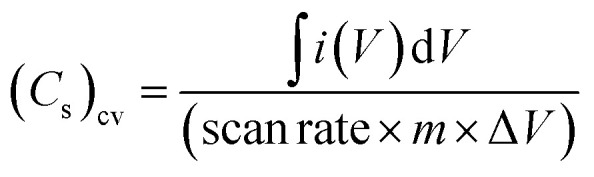
 where ∫*i*(*V*) d*V* is the area under the CV curve, *m* is the mass of active material on one electrode, Δ*V* is the potential window (*V*_2_ − *V*_1_) used for the scan, and the scan rate is the rate at which the potential (voltage) applied to the working electrode is swept over a specified range. For the 10th cycle (2 V, 100 mV s^−1^), the specific capacitance from the CV curve at 35 °C is around ∼142 F g^−1^. Inset of Fig. 10(a) gives the specific capacitance obtains from CV for different voltages.

**Fig. 11:**
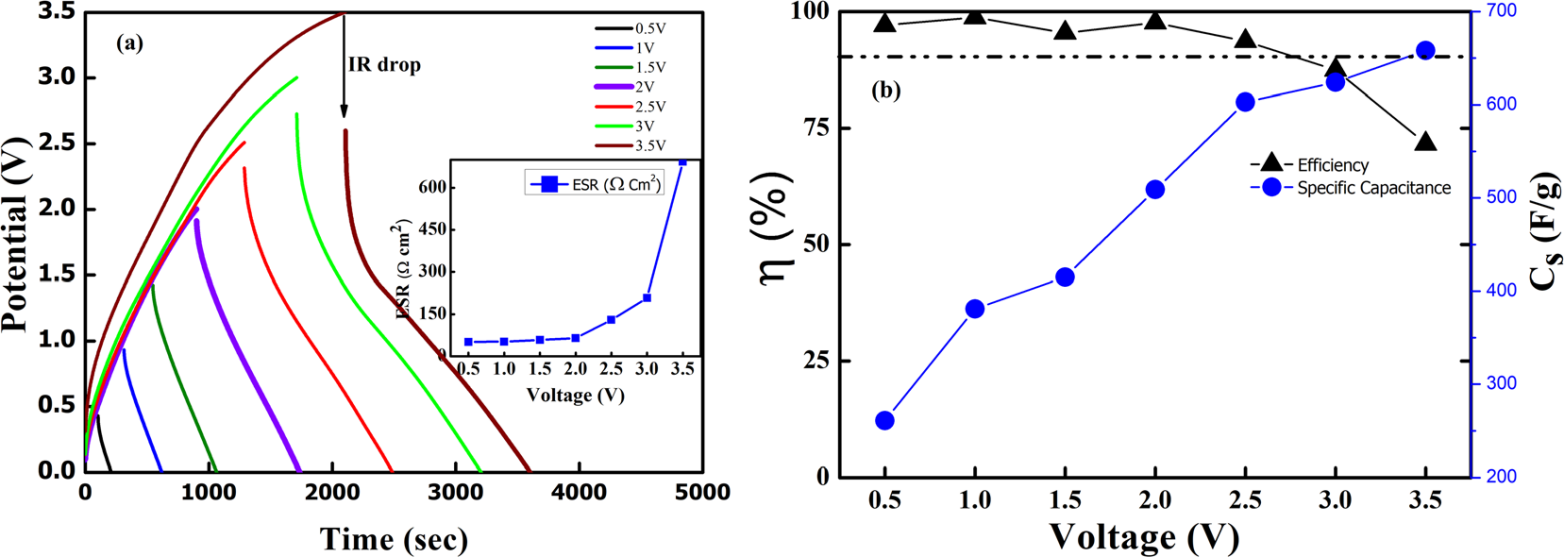
(a) GCD curves to obtain the cutoff voltage at 1 mA discharge current for different potential. (Inset) Nature of ESR with potential (b) coulombic efficiency of the SSC with its specific capacity.

Further, the GCD curves were obtained for different cut-off voltages to evaluate the operating voltage limit, as shown in Fig. 11(a). The IR drop caused by ESR is low for lower potential values. Evidently, beyond ∼2.5 V, the IR drop is quite significant. Inset of Fig. 11(a) shows the variation of ESR with potential. Coulombic efficiency (*η*) was obtained from 
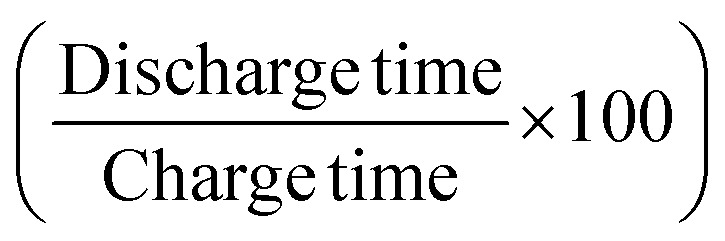
. The ESR values are in agreement with those obtained for the EIS curves. The ESR initially exhibits a low value and increases rapidly above 2.5 V. Fig. 11(b) shows the variation in *C*_s_ and *η* with operating potential. Increasing values of *C*_s_ suggest that the supercapacitor tends to accumulate more charge with increasing cutoff potentials. As a result, the mobile ions can reach close to the pores and electrode interiors for a capacitive action. This allows for an enhanced charge storage capacity, increasing the specific capacitance. Thus, the higher operating potential allows the effective surface area utilization for the mobile ions.^47^ Though *C*_s_ exhibit a monatomic rise, the coulombic efficiency has a slow and gradual drop up to ∼3 V. Beyond that, it reduces sharply to almost 70% at 4 V. Thus, the optimal operating voltage limit for these devices is considered close to 2 V. Importantly, for the voltages ≤2.5 V the GCD cycles are indeed triangular suggestive of EDLC nature. A high specific capacitance is obtained at 2 V (1 mA) of around ∼510 F g^−1^.

Further, the galvanostatic charge–discharge (GCD) cycles were also used to evaluate various performance parameters. The total device capacitance 
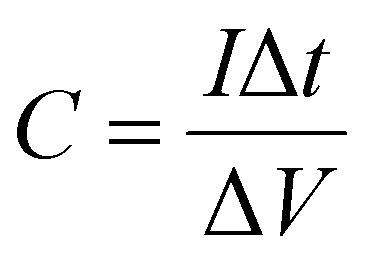
 (in F) is calculated using discharge current (*I* in A), discharge time (Δ*t* in seconds), and voltage window of the discharge cycle (Δ*V* in volts). The specific capacitance (F g^−1^) per electrode has been calculated as 
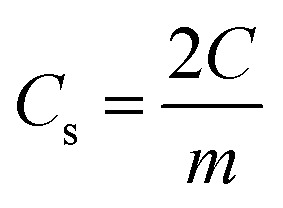
, where *m* is the total activated carbon mass on a single electrode. Moreover, the specific energy 
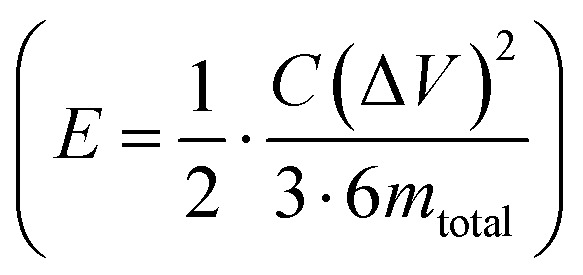
 and specific power 
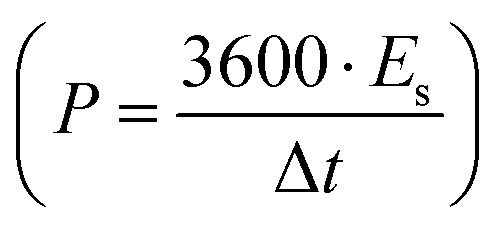
 were obtained for the full device in the units of W h kg^−1^ and W kg^−1^, respectively. Furthermore, the equivalent series resistance 
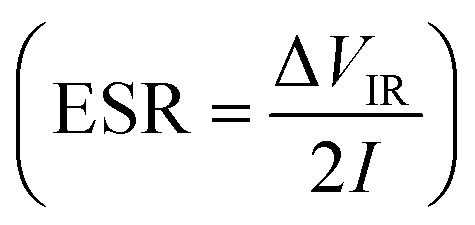
 was calculated using the initial voltage drop during the discharge cycle. For comparison, the areal capacitance values with specific energy and power density were also obtained and given in Table 1. The areal capacitance (F cm^−2^), considering *A* as the cross-sectional electrode area of the device, has been obtained as 
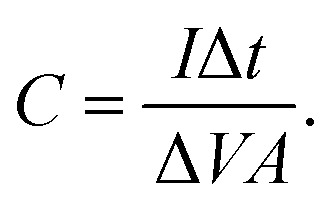
 Further, the energy density (W h cm^−2^) and power density (W cm^−2^) have been calculated as 
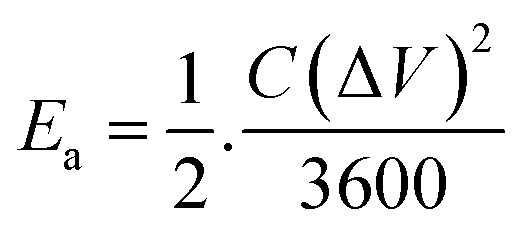
 and 
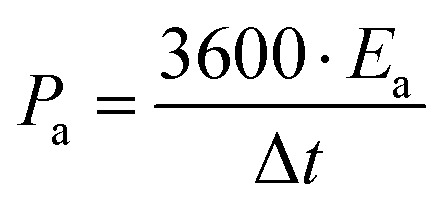
, respectively.

**Table 1:** Specific capacitance (*C*_s_) per electrode, specific energy (*E*), specific power (*P*), equivalent series resistance (ESR), areal capacitance, energy density (*E*_a_), and power density (*P*_a_) for the SSCs of the composite LLTO–(*x*)-EMIM BF_4_ as electrolyte having different composition of *x* = 1, 3, and 6% respectively and operating current and voltage as 2 mA (1.13 A g^−1^) and 2 V respectively. The range is specified for *C*_s_ and (*) represents values for a typical SSC used in the analysis that is close to the average performance of the SSC

LLTO–*x*% EMIM BF_4_	*C*_s_ (F g^−1^)	*E* (W h kg^−1^)	*P* (W kg^−1^)	ESR (Ω cm^2^)	Areal capacitance (mF cm^−2^)	*E*_a_ (μW h cm^−2^)	*P*_a_ (μW cm^−2^)
1	175–185	21*	514*	62*	105*	53*	1298*
	180*						
3	230–240	28*	519*	46*	135*	66*	1225*
	235*						
6	310–320	40*	560*	35*	179*	90*	1242*
	315*						

**Fig. 12:**
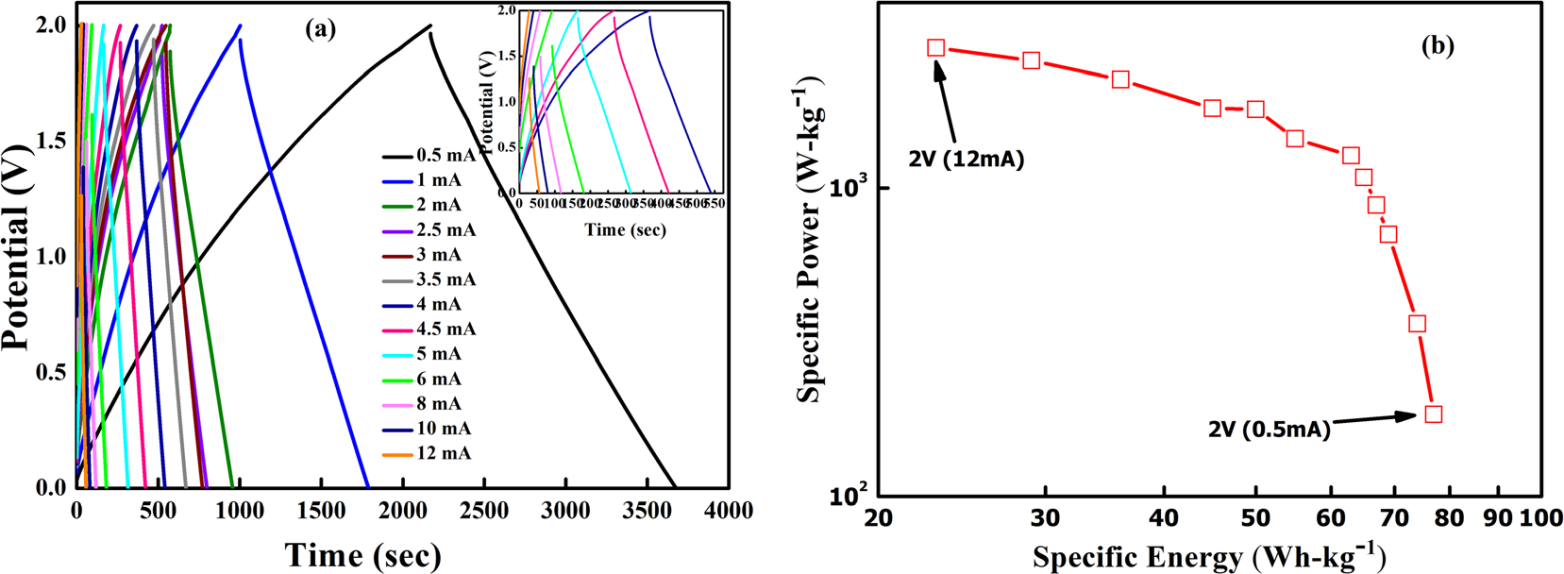
(a) GCD curves of LLTO–6% EMIM BF_4_ for the 10th cycle at different discharge currents ranging from 0.5 mA to 12 mA. (b) Ragone plot for SSC at 0–2 V range.

GCD cycles were then performed at 35 °C for various current densities ranging from 0.5 mA to 12 mA, as shown in Fig. 12(a). Interestingly, the triangular nature is again evident for all discharge currents. Also, IR drop during the discharge cycle reduces gradually as the discharge current decreases. As expected, the discharge time increases as the discharge current decreases. The Ragone plot for the supercapacitor at room temperature within a voltage range of 0–2 V is shown in Fig. 12(b). The specific energy (*E*) and power (*P*) values are appreciable at room temperature. At a low specific power of 184 W kg^−1^, one observes a high specific energy value of 78 W h kg^−1^. The highest specific energy and power were obtained as ∼77 W h kg^−1^ (0.5 mA) and ∼2850 W kg^−1^ (12 mA). Further, with increasing power output, the specific energy decreases.

**Fig. 13:**
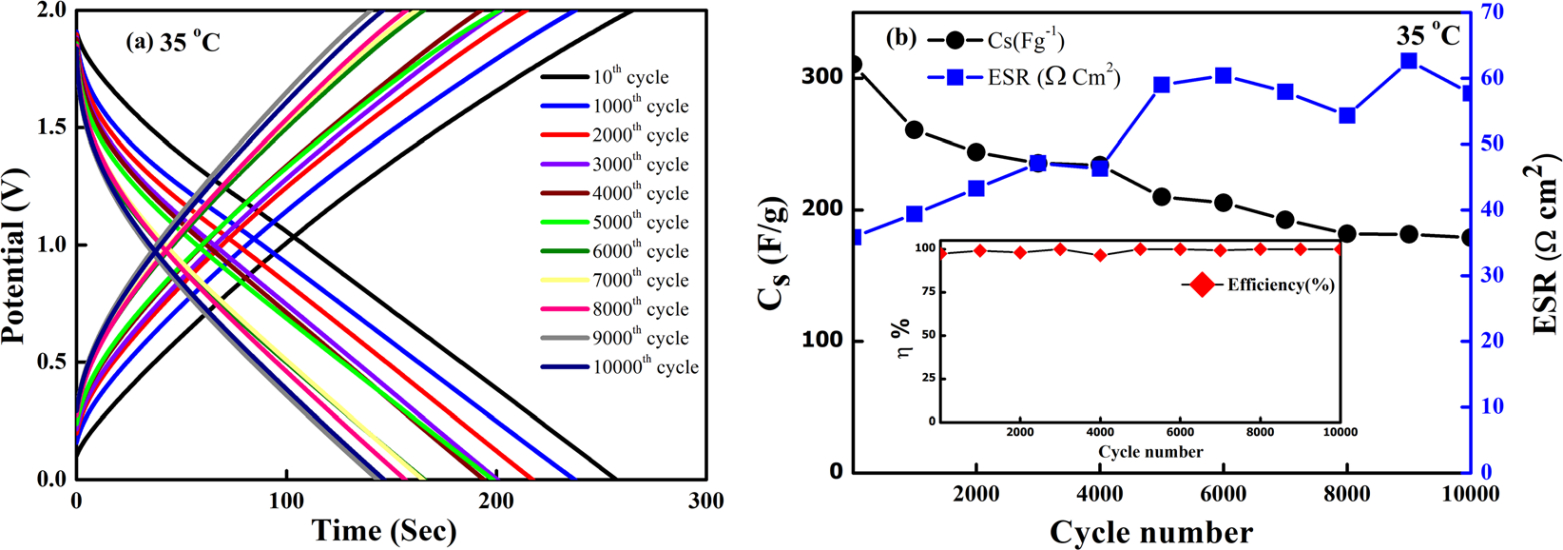
(a) Charge–discharge curves obtained at different cycles for SSC with LLTO–6 EMIM BF_4_ composite as electrolyte at room temperature. (b) Specific capacitance and ESR *vs.* cycle number at room temperature for SSC having electrolyte LLTO–6% EMIM BF_4_. Inset shows coulombic efficiency *vs.* cycle number up to 10 000 cycles.

The SSC device was chosen to operate at an optimized voltage (2 V) and current (2 mA) for long cycling, as the predominant EDLC behaviour is shown below 2.5 V. Fig. 13(a) shows the GCD curve of the SSC. The charge and the discharge curve remain stable even after 10 000 cycles. The *C*_s_ and ESR as a function of the cycle number are shown in Fig. 13(b). The SSC exhibits a good specific capacitance from an initial value of ∼312 F g^−1^ to ∼185 F g^−1^ with ∼60% capacity retention even after 10 000 cycles. The coulombic efficiency of the device remains ∼99% throughout these cycles. The ESR also attains an almost constant value, ranging from (35–57) Ω cm^2^ after 10 000 cycles. Throughout the charging-discharging, up to 10 000 cycles, which in turn shows satisfactory electrochemical stability with a stable electrode–electrolyte interface, no sudden degradation of the device is noticed.

It is interesting to compare the performance parameters of the optimized composition with the other electrolyte compositions as reported in Table 2. Till now, to the best of our knowledge LLTO and its analogues have been successfully applied to batteries. Exploring electrolytic applications to supercapacitor is indeed promising.

**Table 2:** Performance metrics of energy storage device for LLTO as a main compound

Main compound	Composite	Electrical conductivity (Ω^−1^ cm^−1^)	Energy storage applications	Reference
LLTO	LLTO/PVDF HFP	7.7 × 10^−5^	Battery	48
			Specific discharge ∼176 mA h g^−1^	
	LiFePO_4_/LLTO/AC	1.3 × 10^−4^	Battery	49
			Specific energy *E*_s_ ∼2.42 W h kg^−1^	
			Specific power *P*_s_ ∼192 W kg^−1^	
	PVDF-HFP/PPC/LLTO	2 × 10^−4^	Battery	50
			Specific discharge ∼142 A h g^−1^	
	Al–LLTO	3 × 10^−4^	Battery	51
			100-discharge cycle constant capacity 300 mA h g^−1^	
	LLTO + 12.5 BMIM BF_4_	4.7 × 10^−4^	Energy storage	52
	PVDF/PEO/LLTO	3.94 × 10^−3^	Supercapacitors, *C*_s_ ∼182 F g^−1^ long cycling ∼10 000 cycles	53
	LLTO + 6 EMIM BF_4_	∼10^−3^ Ω^−1^ cm^−1^	Supercapacitor	Present investigation
			*C*_s_ ∼510 F g^−1^	
			Good stability, high specific energy and power, coulombic efficiency, remarkable capacitance retention during long cycling	

**Fig. 14:**
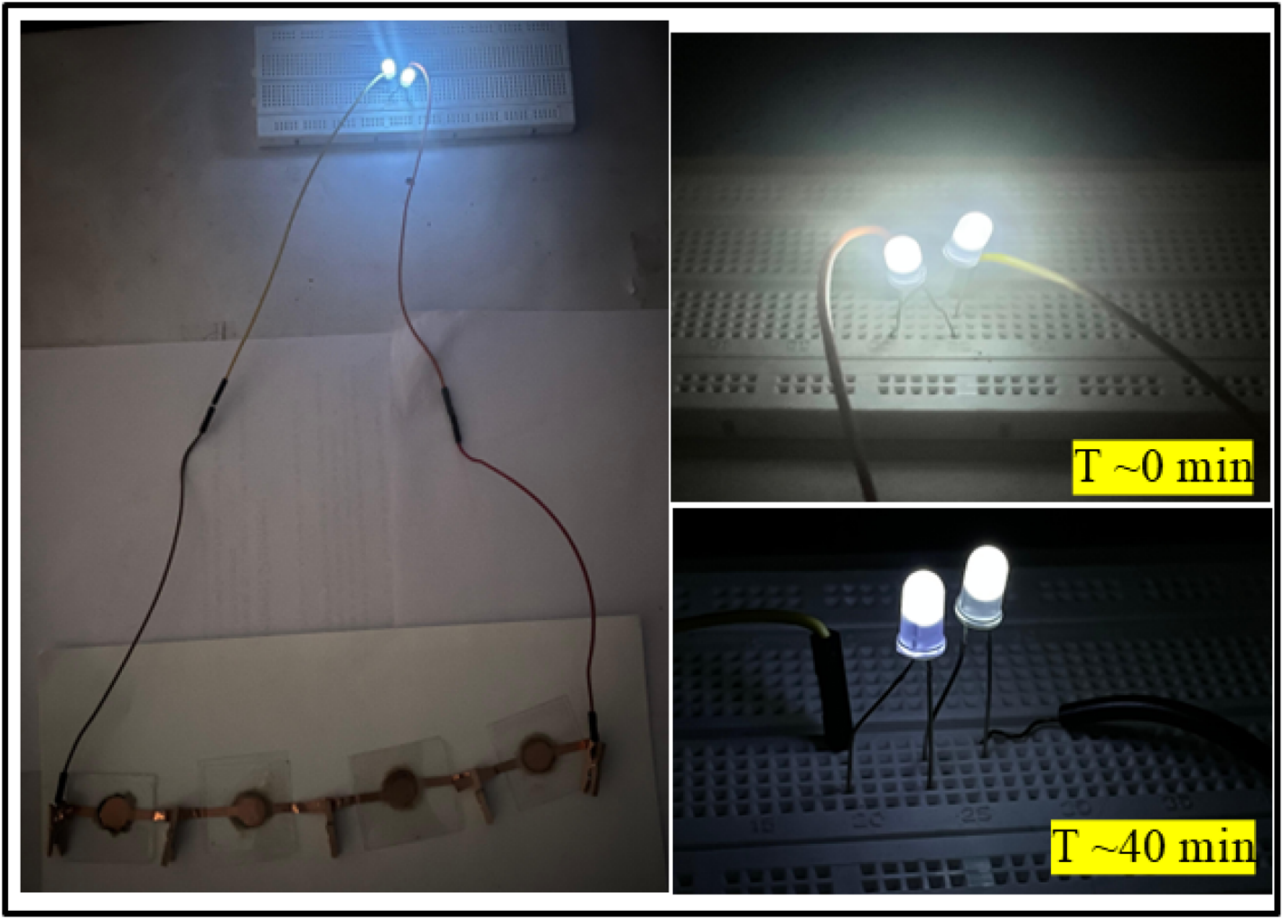
Glow of 3 V white LEDs using four supercapacitor cells connected in series.

To demonstrate the practical applicability of the SSCs, the series combination of four cells having LLTO–6 EMIM BF_4_ composite as an electrolyte has been used to glow two LEDs of ∼6 V as seen in Fig. 14. The LEDs during direct discharge could glow for ∼40 minutes at a temperature of ∼25 °C.

The above discussion may be summarised as follows:

(1) XRD analysis shows the formation of ABO_3_-type perovskite, and even after the addition of the ionic liquid, there is neither shifting of peak nor there is the appearance of a new peak, which readily suggests that the LLTO–IL forms a stable composite without any noticeable chemical reaction. The TGA and FESEM again corroborate the same.

(2) XPS analysis complements the above discussion; even after adding IL LLTO, there is no noticeable shifting of the BE peaks, nor are the present peaks of Li, La, Ti, and O suppressed. Moreover, the Ti element exists in the valence state of Ti^4+^.

(3) The composite 6% EMIM BF_4_–LLTO exhibits the highest room temperature conductivity ∼10^−3^ Ω^−1^ cm^−1^ with the lowest activation energy of ∼0.08 eV, which suggests that the ionic motion of Li^+^-ion is enhanced and facilitated after the addition of the IL in the composite.

(4) The device shows good cycling of ∼10 000 charging–discharging cycles at 2 V (2 mA) with capacity retention of ∼60% after the 10 000 cycles.

## 4 Conclusions

For the first time, solid-state supercapacitors have been developed using perovskite-type Li^+^ ion LLTO. For this, a nominal amount of IL (6% EMIM BF4) was added to the ceramic, and the composition LLTO-*x*-IL (*x* ≤ 6%) was used as an electrolyte for such supercapacitors. Firstly, a high ionic conductivity of ∼10^−3^ Ω^−1^ cm^−1^ at room temperature was achieved by adding a small amount of IL into the LLTO matrix. Further, SSCs were developed by sandwiching the composite mixture between high surface area activated carbon (∼1800 m^2^ g^−1^) electrodes. Even after adding ionic liquid (IL) in the ceramic, the LLTO structure remains unaltered. The XRD, FESEM, TGA, and XPS data corroborated the same. The BET surface area analysis of LLTO indicates that IL occupies the pores and surfaces of LLTO, leading to improved interfacial contacts. The present work investigates SSC performance, given the role of IL. IL provides wettability at the interface, helps intergrain transport, and leads to a better coupling between electrode and electrolyte. For the operating voltage up to 2.5 V, the behaviour of the SSC is predominantly EDLC type, and above this voltage, a pseudo behaviour. The SSCs are stable with time, cycling up to ∼10 000 cycles, and have remarkable capacitance and other performance.

## Data availability

All data included in this work are available upon request by contact with the corresponding author.

## Author contributions

Bhargab Sharma: conceptualization, methodology, validation, formal analysis, investigation, writing – original draft. Shrishti Sharma: formal analysis, Raman analysis, reviewing the original draft. Gurpreet Kaur: formal analysis, reviewing the original draft. Hardeep: formal analysis. Anshuman Dalvi: conceptualization, validation, writing – review & editing, funding acquisition.

## Conflicts of interest

The authors declare that they have no known competing financial interests or personal relationships that could have appeared to influence the work reported in this paper.

## Supplementary Material

RA-015-D4RA07417C-s001
