# Supplementary material for: High energy density solid state symmetric supercapacitors using ionic liquid dispersed Li+ ion-perovskites
Source: RSC Adv. 2025 Jan 27;15(4):2704–16. doi: 10.1039/d4ra07417c (PMC11770677; doi:10.1039/d4ra07417c)
Supplement: RA-015-D4RA07417C-s001 [file RA-015-D4RA07417C-s001.pdf]

Supplementary Information (SI) for RSC Advances.
This journal is © The Royal Society of Chemistry 2025

High Energy density Solid State Symmetric Supercapacitors using Ionic Liquid Dispersed Li^{+} ion-Perovskites

Bhargab Sharma[a], Shrishti Sharma[a], Gurpreet Kaur[b], Hardeep[a], Anshuman Dalvi[a*]

[a] Department of Physics, BITS Pilani-Pilani Campus (RJ-333031) India

[b] Department of Chemistry, BITS Pilani-Pilani Campus (RJ-333031), India

*Corresponding author email: adalvi@pilani.bits-pilani.ac.in

Supplementary Material

1. Tafel Slope analysis

The Tafel slope analysis was conducted for Fig. 10(a). Fig. S1(a) presents the Tafel plots obtained for CV curves for cutoff potentials in a range of 0.5V to 4V. It is noteworthy that the overpotential exhibits a steeper rise at higher current densities. From this plot (as shown in Fig. S1(a) inset, the dotted line) the Tafel slopes were obtained for various cutoff potentials. As apparent in Fig. S1(b), the Tafel slope is almost proportional to the cutoff potential of at least up to 2V, indicative of controlled kinetics. However, as the operating potential increases beyond this point, a partial chemical reaction develops at the interface, further influencing the formation of the electric double-layer capacitance. The Tafel analysis supports findings from other electrochemical workstation analyses, showing predominant electric double-layer capacitor (EDLC) behaviour at least up to 2V. Above this voltage, a slight deviation from the normal ideal (EDLC) behaviour is expected.

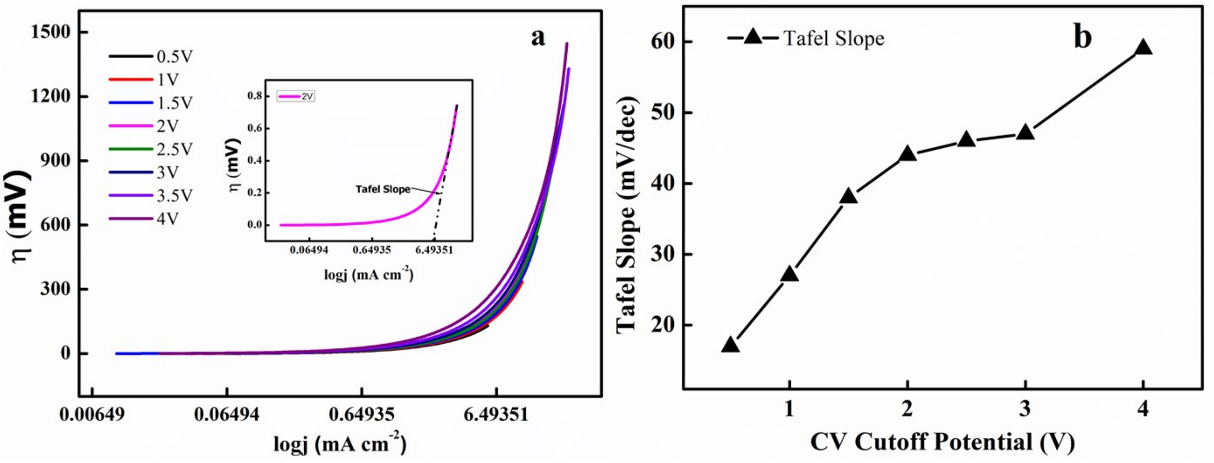

Fig. S1: Tafel slope analysis of cutoff CV potential for **(a)** 0.5V-4V. Inset: Tafel slope for 2V **(b)** Tafel slope vs CV cutoff potential
